# Trimethylamine N-Oxide (TMAO) Impairs Purinergic Induced Intracellular Calcium Increase and Nitric Oxide Release in Endothelial Cells

**DOI:** 10.3390/ijms23073982

**Published:** 2022-04-02

**Authors:** Giulia Querio, Susanna Antoniotti, Federica Geddo, Renzo Levi, Maria Pia Gallo

**Affiliations:** Department of Life Sciences and Systems Biology, University of Turin, 10123 Turin, Italy; giulia.querio@unito.it (G.Q.); susanna.antoniotti@unito.it (S.A.); federica.geddo@unito.it (F.G.); renzo.levi@unito.it (R.L.)

**Keywords:** trimethylamine N-oxide, TMAO, endothelial dysfunction, calcium, nitric oxide, endothelial nitric oxide synthase, vasodilatory mechanism

## Abstract

Trimethylamine N-oxide (TMAO) is a diet derived compound directly introduced through foodstuff, or endogenously synthesized from its precursors, primarily choline, L-carnitine, and ergothioneine. New evidence outlines high TMAO plasma concentrations in patients with overt cardiovascular disease, but its direct role in pathological development is still controversial. The purpose of the study was to evaluate the role of TMAO in affecting key intracellular factors involved in endothelial dysfunction development, such as reactive oxygen species, mitochondrial health, calcium balance, and nitric oxide release using bovine aortic endothelial cells (BAE-1). Cell viability and oxidative stress indicators were monitored after acute and prolonged TMAO treatment. The role of TMAO in interfering with the physiological purinergic vasodilatory mechanism after ATP stimulation was defined through measurements of the rise of intracellular calcium, nitric oxide release, and eNOS phosphorylation at Ser1179 (eNOS^Ser1179^). TMAO was not cytotoxic for BAE-1 and it did not induce the rise of reactive oxygen species and impairment of mitochondrial membrane potential, either in the basal condition or in the presence of a stressor. In contrast, TMAO modified the purinergic response affecting intracellular ATP-induced calcium increase, nitric oxide release, and eNOS^Ser1179^. Results obtained suggest a possible implication of TMAO in impairing the endothelial-dependent vasodilatory mechanism.

## 1. Introduction

Endothelial dysfunction (ED) is a pathological condition, strictly related to thrombosis and atherosclerosis development, characterized by endothelium impairment due to oxidative stress, inflammation and improper balance between vasodilator and vasoconstrictor mediators [1,2,3]. In particular, ED is characterized by endothelial cells’ compromised ability to control the vasodilatory mechanism through nitric oxide (NO) release [3]. In physiological conditions, NO is synthesized by endothelial cells principally through the activation of endothelial nitric oxide synthase (eNOS) by both a calcium-dependent and a calcium-independent mechanism. The first process is activated by the rise of intracellular calcium [Ca^2+^]_i_ due to its entry from extracellular environment or to the opening of intracellular stores during specific agonist stimulation. Ca^2+^–calmodulin interaction activates eNOS which catalyzes the conversion of L-arginine and O_2_ to L-citrulline and NO [4,5]. Endothelial nitric oxide synthase activation can be also mediated by post-translational modifications of the enzyme, such as phosphorylation or dephosphorylation of specific residues. In particular, phosphorylation at Ser1177 (human), or Ser1179 (bovine), causes conformational changes of the enzyme that promote calmodulin interaction with eNOS and the subsequent NO synthesis [4,5,6]. Improper calcium handling in response to extracellular agonists, eNOS Ser1177/Ser1179 dephosphorylation, and reduction of calmodulin binding to eNOS, drive to impairment of NO synthesis. Indeed, impaired activation of eNOS lowers NO bioavailability, thus exacerbating the prevalence of vasoconstrictory mechanisms and favoring vascular damage [1,2].

It has been outlined that ED can be caused by different risk factors, among others, genetic, environmental, and life-style dependent are the most relevant [2,7,8]. In this scenario, diet and its derived metabolites figure as the first, and, at the same time more easily modifiable factors, involved in endothelium impairment. In particular, western diet, rich in animal-derived foods, saturated fats, and simple carbohydrates, has been proposed to be closely related to the onset of endothelial dysfunction and, more generally, of cardiovascular diseases (CVD) [9,10].

Trimethylamine N-oxide (TMAO) is a diet-derived compound, introduced directly through some food matrices or endogenously synthesized from its dietary precursors. Seafood products are examples of direct sources of TMAO, whose concentrations can range from 8 mg to 789 mg/100 g [11]. Choline, L-carnitine, and ergothioneine, abundant in western diet, are the most known TMAO dietary precursors [11]. All the precursors are metabolized by the gut microbiota to form trimethylamine (TMA). In particular, choline, present in meat and dairy products [11], is metabolized by the TMA-lyase and its activating protein S-adenosyl-L-methionine encoded by CutC/D genes [12], while L-carnitine, present in meat [11], is converted to TMA by the oxygenase/reductase system encoded by CntA/B genes. Both choline and L-carnitine can be metabolized by the oxygenase/reductase system, YeaW/X, which has a promiscuous plethora of substrates [12]. Finally, ergothioneine is transformed to TMA by ergothionase [11]. Once TMA is absorbed by enterocytes, it moves to the liver, where it is oxidized by flavin containing monooxygenase 3 (FMO3) to form TMAO [13]. Whether TMAO comes directly from diet or it is endogenously synthesized, it enters the systemic circulation through which it can face different tissues and organs before being excreted through urine [14,15,16]. Even if physiological protective roles of TMAO have been already established for marine animals [17], controversial results have been outlined for the human body, particularly regarding its role in the development of CVD [18]. It is nowadays accepted that higher plasma concentrations of TMAO are present in patients with overt cardiovascular pathology [19,20], but its function as a cause or a marker of disease is still being debated [21,22,23]. Recent evidence outlines a possible role of TMAO in the induction of ED, suggesting a direct role of the molecule in the development of oxidative stress-mediated inflammasome activation both in in vitro and in vivo experiments [24,25,26]. Moreover, several studies highlight a pro-thrombotic potential of TMAO, mediated by an enhancement of platelet responsiveness [27,28].

The aim of the study was to examine the role of TMAO in the onset of endothelial dysfunction. Experiments were performed using bovine aortic endothelial cells (BAE-1). First analyses were addressed to the evaluation of TMAO toxicity, by monitoring cell viability, mitochondrial membrane potential stability and reactive oxygen species increase after acute and prolonged treatments, both in basal conditions and in the presence of a stressor. Further studies were directed to investigate a potential role of TMAO in the impairment of physiological vasodilatory mechanisms, in an extreme condition using the highest plasma concentration of TMAO registered in patients with chronic kidney disease, 100 μM [29,30]. In this context, the effects of TMAO on the purinergic-activated intracellular pathway after ATP stimulation were analyzed by monitoring ATP-induced intracellular calcium variation, nitric oxide synthesis, and eNOS phosphorylation at Ser1179 (eNOS^Ser1179^) in TMAO-treated BAE-1 cells.

## 2. Results

### 2.1. TMAO Does Not Affect Cells Viability

**Figure 1:**
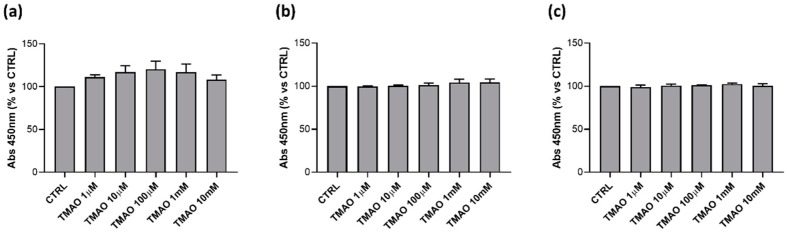
TMAO does not impact BAE-1 viability. BAE-1 were treated with different concentrations of TMAO that showed no cytotoxic effect after (**a**) 24 h, (**b**) 48 h, and (**c**) 72 h (n = 3 independent experiments).

To assess if TMAO has any cytotoxic effect on endothelial cells, BAE-1 were treated with different concentrations (1 μM, 10 μM, 100 μM, 1 mM, and 10 mM) of the compound for 24 h, 48 h, and 72 h. TMAO did not influence cell viability, monitored through the MTS assay, at any time and concentration tested (Figure 1). Because TMAO had no effects on BAE-1 viability, subsequent experiments were performed with a concentration of 100 μM, considered the highest plasma level registered in pathological settings [29,30], both in basal condition and in the presence of stressors.

### 2.2. TMAO Does Not Impair Mitochondrial Membrane Potential

**Figure 2:**
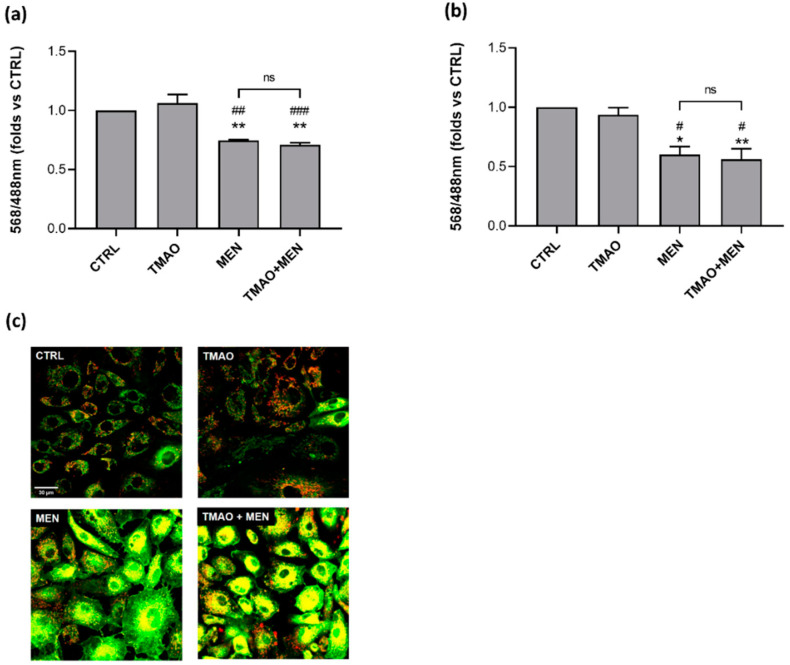
TMAO does not impact mitochondrial membrane potential. Bar graphs of 568/488 nm ratio related to control condition (CTRL) showing that TMAO was not able to induce mitochondrial membrane depolarization after (**a**) 1 h and (**b**) 24 h of treatment and it acted as a neutral factor in presence of a stressor, as MEN, that on the contrary induced a reduction of 568/488 nm ratio. Number of cells for each condition in 1 h treatment: CTRL: 180; TMAO: 180; MEN: 180; TMAO + MEN: 180 (n = 3 independent experiments). Number of cells for each condition in 24 h treatment: CTRL: 83; TMAO: 83; MEN: 83; TMAO+MEN: 83 (n = 3 independent experiments). * *p* < 0.05, ** *p* < 0.01 (vs. CTRL); # *p* < 0.05, ## *p* < 0.01, ### *p* < 0.001 (vs. TMAO). (**c**) Representative confocal microscopy images of BAE-1 treated for 1 h with TMAO 100 μM, MEN 100 μM, MEN 100 μM + TMAO 100 μM. Merged images at 568 nm and 488 nm (magnification 60X).

Mitochondrial membrane potential (∆Ψm) was assessed through confocal microscopy and the ratiometric probe JC-1. Variations of 568/488 nm fluorescence ratio were used as indicators of ∆Ψm depolarization after 1 h or 24 h of treatment with TMAO 100 μM in basal condition or in the presence of Menadione (MEN) 100 μM added in the last hour of TMAO treatment. As Figure 2 shows, TMAO did not induce any effect on ∆Ψm at both times tested, furthermore it did not modify the effect of MEN that, as expected, exerted a reduction in 568/488 nm ratio.

### 2.3. TMAO Does Not Induce the Rise of Reactive Oxygen Species

**Figure 3:**
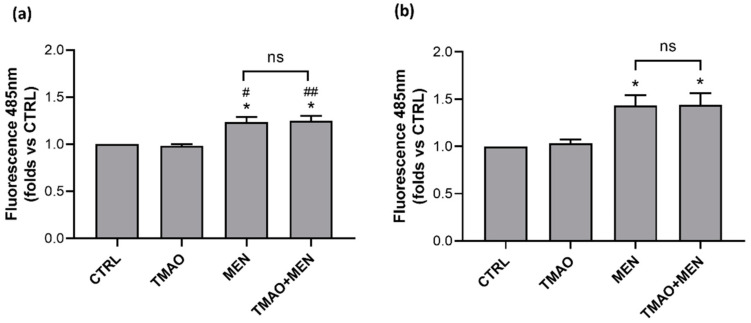
TMAO does not impact reactive oxygen species (ROS) increase. Bar graphs of mean fluorescence (Ex/Em 485 nm/520 nm) related to control condition (CTRL) showing that TMAO 100 μM was not able to induce ROS production in basal condition and it acted as a neutral factor in presence of a stressor, MEN, after (**a**) 1 h and (**b**) 24 h of treatment (n = 3 independent experiments). * *p* < 0.05 (vs. CTRL); # *p* < 0.05, ## *p* < 0.01 (vs. TMAO).

Reactive oxygen species (ROS) increase was monitored through the microplate reader and the CellROX^®^ green probe. Variation in mean cell fluorescence at 485 nm was considered as a predictor of intracellular ROS levels. Data obtained showed similar ROS levels between control condition and TMAO 100 μM treatment both at 1 h and 24 h. Treatment with MEN 100 μM induced, as expected, the rise of ROS, and this effect was maintained in the presence of TMAO (Figure 3).

### 2.4. TMAO Interferes with ATP-Induced Intracellular Calcium Increase

**Figure 4:**
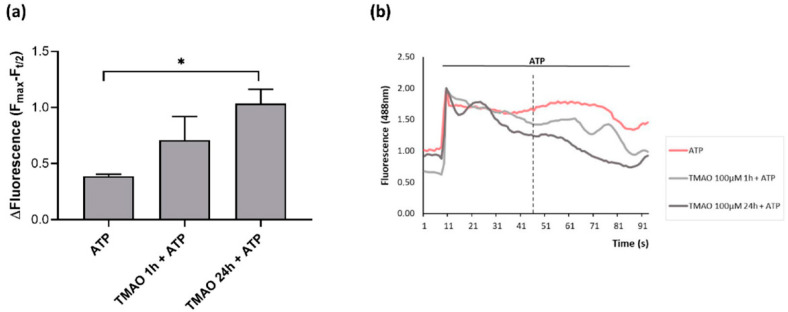
TMAO treatment alters intracellular calcium in purinergic response to ATP. (**a**) Bar graph representing the F_max_-F_t/2_ fluorescence after ATP stimulation in basal condition (ATP) or after treatment with TMAO 100 μM for 1 h or 24 h. Number of cells for each experimental condition: ATP: 47; TMAO 1 h + ATP: 47; TMAO 24 h + ATP: 47 (n = 4 independent experiments). * *p* < 0.05 (**b**) Mean fluorescence variations of normalized curves aligned with respect to the peak value (F_max_) after ATP stimulation in a single representative experiment. Dotted line indicates the t/2 at which curve differences were considered.

Intracellular calcium variations after purinergic stimulation with ATP were monitored in confocal microscopy in time course acquisitions using the Fluo-3 AM probe. As Figure 4a shows, only pretreatment with TMAO for 24 h induced a significant decrease in the calcium signal lengthening with respect to basal ATP stimulation. Intracellular calcium variations were expressed as the difference of the maximal mean fluorescence and the mean fluorescence at t/2 of the total acquisition time (indicated by the vertical dotted line in Figure 4b), (F_max_-F_t/2_), for each experimental condition. Figure 4b presents mean fluorescence curves aligned with respect to the peak value (F_max_) of one representative experiment, in which more pronounced intracellular calcium signal decay is visible in 24 h TMAO-treated cells stimulated with ATP.

### 2.5. TMAO Reduces Nitric Oxide Release in Purinergic Response to ATP

**Figure 5:**
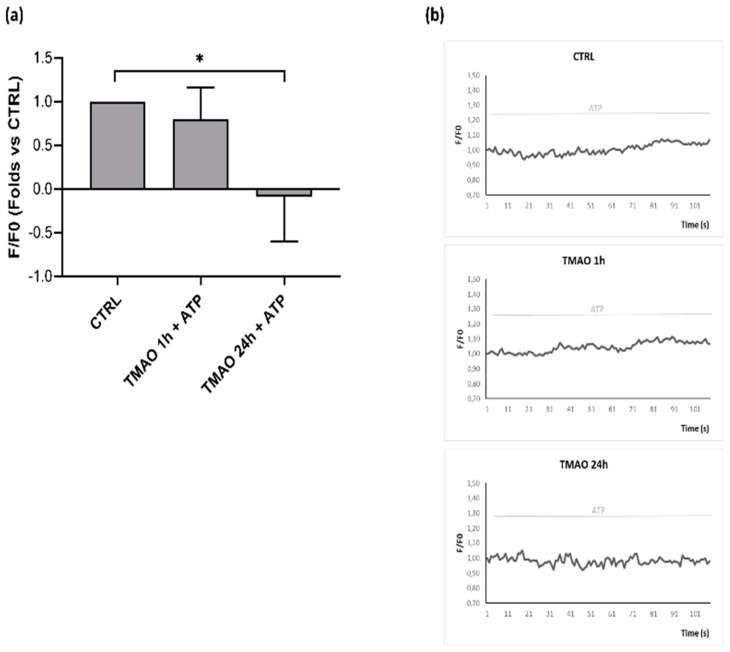
TMAO influences nitric oxide (NO) release in purinergic response to ATP. (**a**) Bar graph showing fluorescence variations before and after ATP stimulation related to basal treatment (CTRL). Treatment with TMAO for 24 h reduced NO release during purinergic stimulation. Number of cells for each experimental condition: CTRL: 63; TMAO 1 h + ATP: 63; TMAO 24 h + ATP: 57 (n = 4 independent experiments). Statistical differences were evaluated with ANOVA and Kruskal–Wallis test. * *p* < 0.05. (**b**) Normalized traces of fluorescence variations (568 nm) of single cells stimulated with ATP in basal condition and after 1 h or 24 h of TMAO pretreatment.

As in endothelial cells, ATP-induced calcium signal triggers eNOS activation and consequently nitric oxide release, thus leading to vasodilation. The next goal was to interlink the inhibitory effect of TMAO on the calcium signal with a TMAO-dependent impairment of ATP-stimulated nitric oxide production. NO release after ATP stimulation was monitored in time course imaging using confocal microscopy and the DAR-4M AM probe. Figure 5a presents fluorescence variations after ATP stimulation and, as the graph shows, only TMAO pretreatment for 24 h induced an alteration in NO release after purinergic stimulation. Figure 5b shows single cell fluorescence variations in a representative experiment, pointing out that ATP stimulation in basal condition and after TMAO pretreatment for 1 h induced fluorescence slope changes, that are directly associated with NO increase. On the contrary, TMAO pretreatment for 24 h inhibited NO release after ATP stimulation, indeed, no fluorescence slope changes were detected.

### 2.6. TMAO Impacts on Endothelial Nitric Oxide Synthase Phosphorylation (eNOS^Ser1179^)

**Figure 6:**
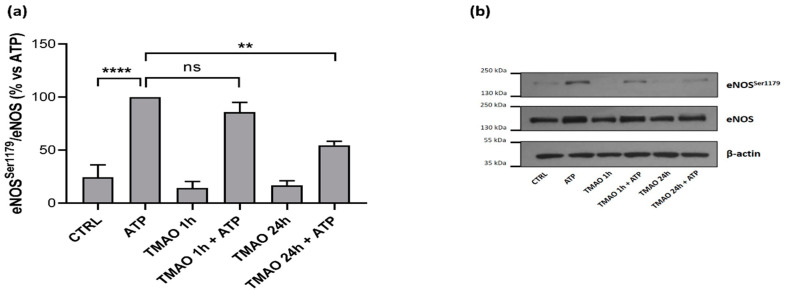
TMAO impacts on eNOS phosphorylation at Ser1179 in purinergic stimulation with ATP. (**a**) Basal treatment with TMAO for 1 h and 24 h did not induce any variation in eNOS phosphorylation, while treatment for 24 h induced a reduction of eNOS^Ser1179^/eNOS ratio in purinergic response to ATP. (n = 4 independent experiments). **** *p* < 0.0001; ** *p* < 0.01. (**b**) Representative Western blot showing the effect of TMAO treatment for 1 h and 24 h in eNOS phosphorylation at Ser1179 after ATP stimulation.

Western blot analyses were performed to evaluate if TMAO could influence eNOS phosphorylation in purinergic response to ATP. As Figure 6a shows, pretreatment of BAE-1 with TMAO for 1 h did not influence the eNOS^Ser1179^/eNOS ratio after purinergic stimulation, while pretreatment with TMAO 100 μM for 24 h before ATP stimulation significantly reduced eNOS phosphorylation, thus suggesting a possible implication of the molecule in the impairment of physiological vascular tone.

## 3. Discussion

ED is a pathological condition characterized by improper balance between vasodilatory and vasoconstrictory mechanisms [1,2,3,7]. Prevention of its development is fundamental to maintain a proper physiological functionality of the cardiovascular system. Several factors have been outlined to favor the development of ED and, among others, genetic, environmental, and life-style dependent are the most studied [2]. In particular, wrong dietary habits can exacerbate a pre-existing unstable condition, thus aggravating oxidative stress and inflammatory response that contribute to the onset of ED [10]. Several studies point out a possible involvement of the diet-derived compound TMAO in cardiovascular diseases development. The aim of the study was to evaluate the role of TMAO in the onset of endothelial dysfunction in an in vitro model using bovine aortic endothelial cells. First results show no cytotoxic effects of TMAO in cells treated for 24 h, 48 h, and 72 h with different concentrations (1 μM, 10 μM, 100 μM, 1 mM, and 10 mM) of the compound (Figure 1). This result is supported by data obtained by Ma et al., that show no reduction in human umbilical vein endothelial cell (HUVEC) viability after 24 h of treatment with TMAO (0, 10, 50, 100 μmol/L) [25]. Further experiments were addressed to the analysis of the TMAO contribution in two conditions that steer towards endothelial damage: impairment of the mitochondrial membrane potential and the rise of reactive oxygen species [1]. The role of MEN in inducing these deleterious effects on endothelial cells has already been studied [31,32], so, in the second part of this work, we focused on the comparison between the impact of TMAO 100 µM, considered the highest plasma concentration registered in patients with chronic kidney disease [29,30], and MEN on ∆Ψm and ROS production. Results presented in Figure 2 and Figure 3 show no effects of TMAO alone in inducing mitochondrial membrane depolarization and reactive oxygen species increase, both after 1 h or 24 h of treatment, and these results are comparable to our previous data obtained in adult rat cardiomyocytes [33]. Furthermore, and according to our report on cardiomyocytes, the effects of MEN were unchanged in cells pretreated with TMAO for 1 h or 24 h [33]. Contrasting results on TMAO and ROS production, obtained by Sun and collaborators, showed increased levels of ROS and lower superoxide dismutase (SOD) activity after HUVEC treatment with TMAO (100, 200, 300 µmol/L) for 1 h, 3 h, and 6 h [26]. These discrepancies could be ascribed to the dissimilarity in the cell model, the setting of time treatments and the TMAO concentration used. Given the lack of changes in redox status and in mitochondrial membrane potential observed in both control and stressed BAE-1 cells treated with TMAO 100 µM, our attention moved towards the potential role of the molecule in affecting the main physiological mechanism of vascular tone regulation, precisely the nitric oxide-mediated pathway. Endothelial dysfunction is indeed strictly related to impaired nitric oxide release. Therefore, the last part of the study was focused on TMAO modulation of the purinergic-dependent intracellular pathway, consisting of the rise of intracellular calcium followed by nitric oxide release, with ATP being used as purinergic agonist. Results obtained show an altered shape of the ATP-induced intracellular calcium signal in cells pretreated with TMAO for 24 h (Figure 4). In particular, we observed significant reduction of the plateau-phase duration. A role of TMAO in the modulation of agonist-induced calcium signals was described by Zhu and coworkers in platelets. Their results showed an increase in IP3-induced calcium release in thrombin-stimulated platelets pretreated with TMAO, and a consequent enhancement of platelet activation and thrombotic event development [27]. Thus, in both studies TMAO seems to affect intracellular calcium homeostasis, even if at different levels, but either way resulting in a detrimental effect on vascular health.

Several cellular proteins have been identified as molecular targets of TMAO with involvement in its pathological effects. Among them, and within the development of vascular dysfunction, proteins of the Nlrp3 inflammasome complex, mitogen-activated protein kinases, protein kinase like ER kinase (PERK), have been recently highlighted. Therefore, a unique and specific molecular mechanism cannot be ascribed to TMAO, and this evidence complies with its general role of protein stabilizer against various stresses. As interestingly proposed by Hong, this property of TMAO could be detrimental in the absence of stressors, as it could affect the conformational flexibility needed for protein functions [34]. In this perspective, the alteration of the ATP-mediated intracellular calcium signal induced by TMAO in our cellular model is time dependent and could be the result of broken conformational changes required both for functionality and mutual interaction of the various calcium channels involved.

As the leading downstream target of purinergic-induced calcium signal in endothelial cells is represented by eNOS, impairment of this key factor could impact NO release. Our results of NO measurement in BAE-1 cells clearly follow this scenario, showing that TMAO treatment for 24 h reduced nitric oxide release (Figure 5). In agreement with these data, treatment with TMAO for 24 h also induced a significant reduction in eNOS^Ser1179^ phosphorylation (Figure 6), thus integrating the whole mechanism leading up to eNOS dysfunctional activity.

A TMAO-dependent reduction in NO release was also observed by Sun et al. after HUVEC treatment with TMAO (100, 200, 300 µmol/L) for 1 h, 3 h, and 6 h [26], even if it was associated with a reduction in eNOS expression rather than in its activation.

On the other hand, a reduction in eNOS phosphorylation was detected in aortic rings from mice fed with a TMAO supplemented diet [24].

The present study shows some limitations, such as the experimental model, that is not based on a human-derived cell line, and the short lasting of TMAO treatment, that is no longer than 24 h and could not be comparable to a chronic exposure, as occurs in some pathological conditions. Despite these weaknesses, different points of strength could be outlined: above all, this is the first in vitro study showing a TMAO mediated impairment of the purinergic dependent NO release in endothelial cells, thus suggesting its direct detrimental effect in the physiological control of vascular tone. Moreover, by using TMAO 100 µM, we aimed to keep close to the highest plasma TMAO concentrations detected in patients with atherosclerotic coronary artery disease and chronic kidney disease [29,30,35]. Furthermore, this last hallmark could explain the ineffectiveness of TMAO in our conditions in affecting ROS levels and mitochondrial health, in comparison with other results obtained with higher TMAO concentrations [36].

In conclusion, this study points out the potential role of TMAO in unsettling the purinergic-activated Ca^2+^- eNOS pathway, adding a new element in the complex scenario of the emergent gut–heart axis concept.

## 4. Materials and Methods

### 4.1. Reagents

Trimethylamine N-oxide (Sigma-Aldrich, Saint Louis, MO, USA) solubilized in culture medium or Tyrode standard solution according to the protocol used, was freshly prepared for each experiment. Menadione (MEN) (Sigma-Aldrich) was solubilized in DMSO at an initial concentration of 100 mM and then diluted in culture medium to the final concentration of 100 μM. ATP was prepared in Tyrode standard solution at a concentration of 10 mM and diluted in culture medium to the final concentration of 100 μM. Tyrode standard solution used in different experiments contained (in mM): 154 NaCl, 4 KCl, 1 MgCl_2_, 5.5 D-glucose, 5 HEPES, 2 CaCl_2_, pH adjusted to 7.34 with NaOH. Unless otherwise specified, all reagents for cell culture and experiments were purchased from Sigma-Aldrich.

### 4.2. Cell Culture

Bovine aortic endothelial cells-1 (BAE-1, ECACC, Salisbury, UK) maintained in Dulbecco’s Modified Eagle Medium (DMEM) 1 g/L glucose supplemented with 10% heat-inactivated FBS, 2 mM L-glutamine, and 50 μg/mL gentamycin, were incubated at 37 °C in a humidified atmosphere containing 5% CO_2_ during all experiments. BAE-1 were used from passage 2 to 7.

### 4.3. Cell Viability

Assessment of cell viability was performed with CellTiter 96^®^ AQueous One Solution Cell Proliferation Assay, using the tetrazolium compound 3-(4,5-dimethylthiazol-2-yl)-5-(3-carboxymethoxyphenyl)-2-(4-sulfophenyl)-2H-tetrazolium, inner salt (MTS, Promega, Madison, WI, USA). The assay is based on the bioreduction by metabolically active cells of MTS into a colored formazan product, soluble in tissue culture medium. BAE-1 were seeded (3.3 × 10^4^ cells/200 μL/ well) into a 96-well plate in culture medium and incubated at 37 °C for 24 h. Following incubation and cell adhesion, BAE-1 were treated for 24 h, 48 h, and 72 h with different concentrations of TMAO (1 μM, 10 μM, 100 μM, 1 mM, 10 mM). Three hours before the end of the treatment MTS (20 μL/100 μL) was added in each well. Formazan product formation was measured with FilterMax F5TM Multi-Mode microplate reader (Molecular Devices, Sunnyvale, CA, USA) at 450 nm, and the detected absorbance was considered proportional to the number of viable cells. Data from three independent experiments were expressed as mean percentage referring to the control.

### 4.4. Mitochondrial Membrane Potential

Mitochondrial membrane potential (∆Ψm) was monitored with confocal microscopy and the 5,5′,6,6′-tetrachloro-1,1′,3,3′-tetraethyl-imidacarbocyanine iodide (JC-1) probe. JC-1 is a ratiometric indicator of ∆Ψm; it is able to shift its fluorescence from red to green when a depolarization of the mitochondrial membrane occurs. BAE-1 were seeded at a density of 3.3 × 10^4^ cells/mL on uncoated glass bottom dishes of 35 mm diameter (Ibidi, Martinsried, Germany) in culture media and incubated at 37 °C for 24 h. Following incubation, cells were treated according to experimental conditions: TMAO 100 μM for 1 h, MEN 100 μM for 1 h, TMAO 100 μM + MEN 100 μM for 1 h, for acute stimulation tests, and TMAO 100 μM for 24 h, MEN 100 μM for 1 h, TMAO 100 μM for 24 h + MEN 100 μM for 1 h, for prolonged stimulation tests. JC-1 probe (10 μM) was added in each dish 30 min before the end of the treatment and cells were then washed three times with PBS containing Ca^2+^ and Mg^2+^ to avoid cell loss. Fluorescence intensity variations were measured at 568 nm and 488 nm through an Olympus Fluoview 200 laser scanning confocal system (Olympus America Inc., Melville, NY, USA) mounted on an inverted IX70 Olympus microscope, and all acquisitions were performed with a 60X Uplan FI (NA 1.25) oil-immersion objective. ∆Ψm was calculated through the red/green fluorescence ratio and the definition of the regions of interest (ROIs) using the software ImageJ (Rasband, W. S., ImageJ, U. S. National Institutes of Health, Bethesda, MD, USA; https://imagej.nih.gov/ij/ (accessed on 7 February 2022), 1997–2017) and expressed as the mean value of the resulting ratios for each condition compared to the control of four independent experiments.

### 4.5. Reactive Oxygen Species

Reactive oxygen species (ROS) variations were monitored through the microplate reader and the CellROX^®^ green probe (Thermo Fisher Scientific, Waltham, MA, USA). BAE-1 were seeded (3.3 × 10^4^ cells/200 μL/well) on black clear bottom 96-well plates (Greiner Bio-One, Kremsmünster, Austria) and incubated at 37 °C for 24 h. Following incubation, cells were treated according to experimental conditions: TMAO 100 μM for 1 h, MEN 100 μM for 1 h, TMAO 100 μM + MEN 100 μM for 1 h, for acute stimulation tests, and TMAO 100 μM for 24 h, MEN 100 μM for 1 h, TMAO 100 μM for 24 h + MEN 100 μM for 1 h, for prolonged stimulation tests. CellROX^®^ green (5 μM) was added in each well 30 min before the end of the treatment and cells were then washed twice with PBS containing Ca^2+^ and Mg^2+^ to avoid cell loss. Fluorescence intensity variations were measured at 485 nm through the FilterMax F5TM Multi-Mode microplate reader. ROS production was expressed as mean fluorescence compared to the control of three independent experiments.

### 4.6. Intracellular Calcium in Response to ATP Stimulation

Intracellular calcium variations were monitored through time course acquisitions in confocal microscopy and Fluo-3 AM probe (Thermo Fisher Scientific). BAE-1 were seeded at a density of 3.3 × 10^4^ cells/mL on uncoated glass bottom dishes of 35 mm diameter (Ibidi) in culture media and incubated at 37 °C for 24 h. Following incubation, cells were treated for 1 h or 24 h with TMAO 100 μM or were left in culture medium. Fluo-3 AM (2 μM) was added in each dish 30 min before the end of all treatments and cells were then washed twice with Tyrode standard solution. Fluorescence intensity variations in time were measured at 488 nm through an Olympus Fluoview 200 laser scanning confocal system mounted on an inverted IX70 Olympus microscope, and all acquisitions were performed with a 60X Uplan FI (NA 1.25) oil-immersion objective. During the experiments BAE-1 were maintained in Tyrode standard solution containing or not TMAO according to pretreatment protocols; ATP 100 μM and ATP 100 μM + TMAO 100 μM used for stimulation were added through a microperfusion system (pipette diameter 200 μm). Intracellular calcium variations in time were analyzed through the definition of the ROIs, using the software ImageJ. Changes in intracellular calcium concentration were calculated as F/F_0_ to normalize the traces; moreover, an additional analysis was applied in order to highlight changes in the ATP-induced calcium signal profile between the control and TMAO-treated cells. First, traces of the same dish were mediated; from each of four independent experiments, mean traces from every condition (ATP, TMAO 1 h + ATP, TMAO 24 h + ATP) were aligned with respect to the peak value (F_max_). Then, the difference between F_max_ and mean fluorescence at t/2 (F_t/2_), (F_max_-F_t/2_), of the aligned curves was compared in order to evaluate if any perturbation in calcium curves occurred.

### 4.7. Nitric Oxide Release after ATP Stimulation

Nitric oxide (NO) release was monitored through time course acquisitions in confocal microscopy and DAR-4M AM probe (Thermo Fisher Scientific). BAE-1 were seeded at a density of 3.3 × 10^4^ cells/mL on uncoated glass bottom dishes of 35 mm diameter (Ibidi) in culture media and incubated at 37 °C for 24 h. Following incubation cells were treated for 1 h or 24 h with TMAO 100 μM or were left in culture medium. DAR-4M AM (5 μM) was added in each well 30 min before the end of the treatment and cells were then washed twice with Tyrode standard solution. Fluorescence intensity variations in time were measured at 568 nm through an Olympus Fluoview 200 laser scanning confocal system mounted on an inverted IX70 Olympus microscope, and all acquisitions were performed with a 60X Uplan FI (NA 1.25) oil-immersion objective. During the experiments BAE-1 were maintained in Tyrode standard solution containing or not TMAO according to pretreatment protocols; ATP 100 μM and ATP 100 μM + TMAO 100 μM used for stimulation were added through a microperfusion system (pipette diameter 200 μm). NO release variations in time were analyzed through the definition of the ROIs using the software ImageJ. NO release after different treatments was expressed as percentage change in mean fluorescence detected ((F_max_ − F_0_)/F_0_) × 100 for each trace. Normalized percentage change values of three independent experiments were then mediated and expressed as folds toward basal ATP treatment to verify if any variation in NO release could be detected in different treatments.

### 4.8. Western Blot

Total eNOS and its phosphorylated form (eNOS^Ser1179^) in purinergic stimulation with ATP 100 μM for 1 min after treatment with TMAO 100 μM for 1 h or 24 h were monitored through Western blot. BAE-1 were seeded at a density of 4 × 10^4^ cells/mL on plastic petri dishes with 22.1 cm^2^ of growth area in culture media and incubated at 37 °C for 24 h. Following incubation, cells were treated for 1 h or 24 h with TMAO 100 μM or were left in culture medium. After the treatment with ATP 100 μM for 1 min, cells were lysed with RIPA lysis buffer (ThermoFisher Scientific) containing phosphatase inhibitor cocktail (PhosSTOP, Roche, Mannheim, Germany), forced through a 1 mL syringe needle several times, centrifuged at 10,000 rpm for 5 min at 4 °C and stored at −80 °C. Protein lysates (20 μg per lane) were run on 8% SDS-PAGE gel, transferred to a polyvinylidene fluoride membrane (PVDF) and blocked for 1 h in TBST (10 mM Tris–HCl, pH 7.5, 0.1 M NaCl, 0.1% Tween 20) plus 5% non-fat dry milk at 37 °C. PVDF were incubated overnight at 4 °C with primary antibodies (monoclonal anti-eNOS, 1:500 dilution, BD, Biosciences; polyclonal anti-eNOS^Ser1179^, 1:250 dilution, Thermo Fisher Scientific; monoclonal anti-β-actin, 1:2000 dilution, Sigma-Aldrich). Membranes were then washed three times with TBST and incubated for 1 h at room temperature with horseradish peroxidase-conjugated secondary antibodies (anti-mouse, 1:20,000 dilution, Amersham, for total eNOS and β-actin; and anti-rabbit, 1:10,000 dilution, Amersham, for eNOS^Ser1179^) and washed again three times with TBST. Protein bands were localized by chemiluminescence with Western Lightning Plus-ECL (Perkin Elmer, Waltham, MA, USA). Protein levels were determined using the software ImageJ and expressed as mean percentage toward ATP condition of eNOS^Ser1179^/eNOS ratio of four independent experiments.

### 4.9. Statistical Analysis

Data are presented as mean ± SEM. All data were analyzed with GraphPad Prism 8.0.1 software using ANOVA followed by Bonferroni’s multiple comparison for post hoc tests. Differences with *p* < 0.05 were considered statistically significant.

## Data Availability

Not applicable.
